# Diet Quality and Nutritional Risk Based on the FIGO Nutrition Checklist among Greek Pregnant Women: A Cross-Sectional Routine Antenatal Care Study

**DOI:** 10.3390/nu15092019

**Published:** 2023-04-22

**Authors:** Maria G. Grammatikopoulou, Meletios P. Nigdelis, Anna-Bettina Haidich, Maria Kyrezi, Helga Ntine, Maria Papaioannou, Gesthimani Mintziori, Dimitrios P. Bogdanos, George Mavromatidis, Dimitrios G. Goulis

**Affiliations:** 1 Unit of Immunonutrition and Clinical Nutrition, Department of Rheumatology and Clinical Immunology, Faculty of Medicine, School of Health Sciences, University of Thessaly, Biopolis, GR-41110 Larissa, Greece; bogdanos@med.uth.gr; 2 Unit of Reproductive Endocrinology, 1st Department of Obstetrics and Gynecology, Papageorgiou General Hospital, Aristotle University of Thessaloniki, GR-56403 Thessaloniki, Greece; meletis.nigdelis@gmail.com (M.P.N.); mariakir2000@gmail.com (M.K.); gefsi@auth.gr (G.M.); dgg@auth.gr (D.G.G.); 3 Klinik für Frauenheilkunde, Geburtshilfe und Reproduktionsmedizin, Universitätsklinikum des Saarlandes, Gebäude 9, Kirrberger Straße, DE-66421 Homburg, Germany; 4 Department of Hygiene, Social-Preventive Medicine & Medical Statistics, School of Medicine, Faculty of Health Sciences, Aristotle University of Thessaloniki, University Campus, GR-54124 Thessaloniki, Greece; haidich@auth.gr; 5 2nd Academic Department of Obstetrics and Gynaecology, Hippokration General Hospital, Aristotle University of Thessaloniki, 49 Konstantinoupoleos Street, GR-54642 Thessaloniki, Greece; cldiatrofi@gmail.com (H.N.); maria-pap27@hotmail.com (M.P.); mavromag@auth.gr (G.M.)

**Keywords:** gestation, triage, MUAC, nutritional screening, pregnancy, nutritional assessment, malnutrition, vitamin D, folic acid, gestational diabetes mellitus

## Abstract

The International Federation of Gynecology and Obstetrics (FIGO) nutrition checklist is a tool for everyday antenatal clinical practice, easy to use by most healthcare professionals, aiming to initiate a conversation regarding gestational weight gain (GWG) and nutrition and identify women who might require further assessment. The present cross-sectional study aimed to apply the FIGO nutrition checklist to pregnant women attending routine antenatal care and identify nutritional risk factors. Pregnant women (*n* = 200) were recruited from the outpatient pregnancy clinics of two hospitals in Thessaloniki and completed the checklist. The FIGO-diet quality score and the FIGO-nutritional risk score (NRS) were calculated. The results revealed that 99% of the women exhibited at least one nutritional risk factor based on the checklist. The median FIGO diet quality score of the sample was 4.0 (3.0–5.0), with 95% of the participants responding negatively to at least one question, indicating the need for improving diet quality. Improved diet quality was noted in cases of hyperemesis gravidarum and among those receiving vitamin D supplements. A large percentage of the participants (36%) exhibited five or more nutritional risk factors, as indicated by a total FIGO-NRS below 5. Women with low middle-upper arm circumference, indicative of protein-energy malnutrition (20.6% of the sample), exhibited more nutritional risk factors compared with the rest. On the other hand, being in the third trimester of pregnancy was associated with lower nutritional risk and, subsequently, better diet quality.

## 1. Introduction

Maternal nutrition is a quintessential element of optimal obstetrical and birth outcomes [1,2,3] and an important effector of offspring [4,5,6,7,8,9] and maternal health [7,10,11]. Since nutrition is a modifiable factor, pregnancy can be viewed as an opportunity to improve diet quality and nutritional status and reduce the possibility of developing adverse events. In parallel, dietary interventions performed during the pre-gestational and gestational periods will likely be cost-effective, lowering the projected economic burden [12]. Although women report improvements in their diet after conception is achieved [13], in their majority, they fail to adhere to the dietary recommendations, often exhibiting micronutrient deficiencies [12,14,15,16,17,18]. In parallel, societal factors paired with the high demands of pregnancy pose women with greater nutritional risks than men. The marked increase in the prevalence of overweight and obesity among women of reproductive age, nutritional inequalities, food insecurity, and maternal malnutrition, in particular iron deficiency anemia (IDA) [17,19,20,21,22], have collectively contributed to the prioritization of maternal nutrition by the World Health Organization (WHO) [23].

Maternal nutrition has also gained significance on the agendas of scientific societies with the development of nutrition recommendations and tools for assessing nutritional adequacy and appropriate gestational weight gain (GWG) [24,25,26]. However, apart from body weight measurements, the prescription of oral nutrient supplementation (ONS), and the assessment of hemoglobin (Hb) concentrations, health care practitioners (HCPs)—including midwives, obstetricians and gynecologists (OBGYNs), registered dietitians and nutritionists (RDNs), and general practitioners (GPs)—often do not have the time, skills, or confidence to triage pregnant women for further nutrition education and counseling [27].

More recently, the International Federation of Gynecology and Obstetrics (FIGO) Adolescent, Preconception, and Maternal Nutrition Working Group developed a short and simple checklist [28], evaluating diet quality and nutritional risk factors in the preconception and gestational periods based on the “Think nutrition first” FIGO report [29]. The FIGO nutrition checklist [28] consists of four domains with 12 questions in total, evaluating (i) the need for special dietary requirements, (ii) GWG, (iii) diet quality, and (iv) the possible need for oral nutrient supplementation (ONS). The checklist takes 5–10 min to complete [30] and is the only tool for assessing maternal nutritional risk factors. It is simple to use [30], suitable in both low-middle and high-income settings [31], and can be administered by HPCs who are not nutrition-savvy to support standardized and optimal antenatal care and include nutritional assessment in routine practice. The checklist is accompanied by a supportive document summarizing the nutrition recommendations [32]. It has already been translated into Spanish and French, and aside from the printed version [28], it is also available for online completion [33].

The present cross-sectional study aimed to apply the FIGO nutrition checklist to pregnant women attending routine antenatal care in Greece, identify nutritional risk factors, and review the scientific literature using the FIGO nutrition checklist to date.

## 2. Materials and Methods

### 2.1. Population Recruitment

A total of 230 pregnant women were recruited from the outpatient pregnancy clinics of the 1st and 2nd Departments of Obstetrics and Gynecology, situated at Papageorgiou and Hippokration General Hospitals, respectively, in Thessaloniki, northern Greece. Recruitment took place from December 2021 until February 2023.

Inclusion criteria involved (i) adult pregnant women, (ii) women who were willing to participate, and (iii) those able to understand the Greek language and communicate effectively. Exclusion criteria involved (i) adolescent pregnant women, (ii) not consenting to participate, or (iii) being unable to understand the Greek language and communicate effectively.

Missing data were apparent in 30 participants; thus, the final sample consisted of 200 pregnant women, with their characteristics being presented in Table 1.

### 2.2. Ethics Approval

The study was conducted under the Declaration of Helsinki and approved by the Institutional Review Boards of the Hippokration General Hospital (49610/02-11-2021 and 4528/30-01-2023) and the Papageorgiou General Hospital (353/13-10-2022) of Thessaloniki. All women provided informed consent prior to participation.

### 2.3. Translation of the FIGO Nutrition Checklist

The FIGO nutrition checklist was translated into the Greek language with the kind permission of Dr Sarah Louise Killeen (personal communication) on behalf of the FIGO. The four-step forward-backward process was applied, as proposed by Guillemin et al. [35]. The final translated tool is presented in Supplementary Material.

### 2.4. Procedures

Pregnant women visiting the outpatient clinics of the hospitals mentioned above were asked to fill in the FIGO checklist [28] with the help of experienced RDNs (H.N., M.P., or M.K.). Participant characteristics (age, marital status, educational level, and income) and pregnancy characteristics of the pregnancy (trimester, singleton/twin, gravidity, method of conception, hyperemesis gravidarum, gestational diabetes mellitus [GDM], preeclampsia, or hypertensive disorders of pregnancy diagnoses) were also recorded with the help of the OBGYNs.

#### 2.4.1. Anthropometric Indices

Body weight was measured with a Seca 700 mechanical scale (Seca, Hamburg, Germany), with the subjects dressed in minimal clothing. Height was measured with a wall-mounted Harpenden stadiometer (Holtain, Crymych, UK) with the subjects barefoot. All anthropometric measurements were performed in the morning by experienced dietitians. The subjects reported their pre-gravid body weight.

Pre-pregnancy body mass index (BMI) was calculated as each participant’s body weight (kg) divided by their height squared (m^2^). GWG was calculated for each participant according to the pre-gravid BMI and according to the Institute of Medicine (IOM) thresholds [34].

Middle-upper arm circumference (MUAC) was also measured in all participants using an elastic tape, and the WHO cut-offs were applied for the diagnosis of undernutrition (<23 cm) or overnutrition (≥33 cm) [24].

#### 2.4.2. Hemoglobin Concentrations

Hb concentrations were collected from the electronic records of each participant for the same gestational time-point as the completion of the FIGO nutrition checklist.

### 2.5. Calculation of Scores from the FIGO Nutrition Checklist

Questions on the FIGO nutrition checklist [28] were divided into two parts. The first part consisted of six dietary intake questions presented as a short food frequency questionnaire (FFQ). These were used to calculate the FIGO-diet quality score, as suggested by Tsoi and associates [36]. Each positive response (Yes) on the six FFQ questions was awarded one point, whereas negative responses were given no points [36]. The total diet quality score was calculated from the points received in these six questions. According to Tsoi [36], the score (a continuous variable) may range from 0 to a maximum of 6, with higher scores indicative of better diet quality. The variable was analyzed as a continuous variable.

In parallel, the six dietary intake questions and the three oral nutrient supplementation (ONS) questions (parts 3 and 4) of the checklist were used for the calculation of the total nutritional risk score (NRS) based on the FIGO nutrition checklist [28]. The FIGO-NRS ranges between 0 and 9, with lower scores indicating more nutritional risk factors and the need for nutritional counseling.

### 2.6. Statistical Analyses

Continuous variables were assessed for normality using the Shapiro–Wilk test and visual inspection of the data (histograms). Given that all variables were non-normally distributed, they were presented as medians with their respective interquartile ranges. Qualitative variables were summarized as absolute counts with their respective frequencies (percentages).

Differences in the nutritional score were assessed using the Mann–Whitney test when differences between two groups were examined, and the Kruskal–Wallis test when more than two groups were assessed. When the FIGO NRS scores were compared between participants of different gestational trimesters, Dwass–Steel–Critchlow–Fligner pairwise comparisons were undertaken. Regarding MUAC categories, violin plots were used to describe NRS distribution among the three distinct nutritional status categories (malnourished, normal, and adipose). Furthermore, differences in the percentages of pregnant women responding to at least one negative answer in the FIGO Nutrition Checklist between different response groups were assessed using the chi-squared test.

Univariable logistic regression was used to assess the relationship between the FIGO-Diet Quality Score > 3 and parameters related to obstetric history and nutrition status. The odds ratio (OR) and 95% confidence intervals (CI) were calculated, along with the *p*-value of the Wald test. Following univariable models, a multivariable model was fitted with all examined variables and another containing only the statistically significant ones.

Furthermore, exploratory binomial univariable logistic regression was performed to evaluate the association between high overall nutritional risk (based on questionnaire responses) and clinical parameters related to nutrition among pregnant women. Given the absence of specific thresholds for the overall score, pregnant women were divided into a high (FIGO-NRS < 5) and a low nutritional risk group (FIGO-NRS ≥ 5). A multivariable model was fitted based on all variables (given their clinical significance) to examine associations between clinical parameters.

Analyses were conducted using Jamovi (Version 2.3.21.0), while figures were constructed using the *ggplot2* package in R Studio (Version 2022.12.0 + 353). Statistical significance was considered in cases of two-sided *p*-values lower than 0.05.

Missing data referred to absent responses to specific questions. No manipulations were conducted for missing data, as this reached a maximum of 1% per variable (two missing values in 200 pregnant women). No manipulations were conducted for multiple comparisons [37].

## 3. Results

### 3.1. Diet Quality and Nutritional Risk Based on the FIGO Nutrition Checklist

**Figure 1:**
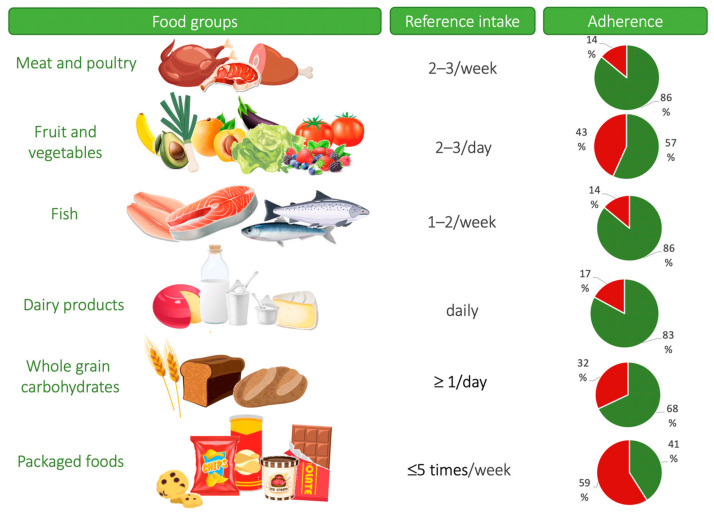
Percentage (%) of positive (green) and negative (red) answers provided by pregnant Greek women (*n* = 200) regarding the components of the FIGO-Diet quality. FIGO—International Federation of Obstetrics and Gynecology.

The results revealed that 99% of the participating pregnant women had at least one nutritional risk factor based on the FIGO nutrition checklist (Table 2). The median FIGO diet quality score of the sample was 4.0 (3.0–5.0), with 95% of the participants responding negatively to at least one question, indicating the need to improve diet quality. Most negative answers (59.5%) involved the consumption of packaged foods, which exceeded in frequency the five-times weekly threshold suggested by FIGO. Most positive answers (86%) involved the frequency of consumption of meat and poultry (2–3 times per week). Positive FIGO nutrition checklist components regarding the quality of the diet as reported by the present sample are also presented in Figure 1 in a more graphical manner.

Table 3 details the components of the FIGO nutrition checklist for each trimester of pregnancy. No differences were noted in the intake of meat/poultry, fruit and vegetables, fish, dairy products, or pre-packaged foods among participants in different gestational trimesters. ONS with folate increased with each ascending gestational trimester, and so did Hb concentrations within the normal range. The FIGO diet quality score was indifferent between women in different pregnancy trimesters; however, the FIGO-NRS increased with ascending trimesters. After adjustment of the *p*-value for multiple comparisons (at 0.016), differences were demonstrated between the 1st and 3rd trimesters (*p* < 0.001), as well as between the 2nd and 3rd trimesters (*p* = 0.012) regarding the FIGO-NRS score. The differences between the first and second trimesters were not significant (*p* = 0.28).

**Figure 2:**
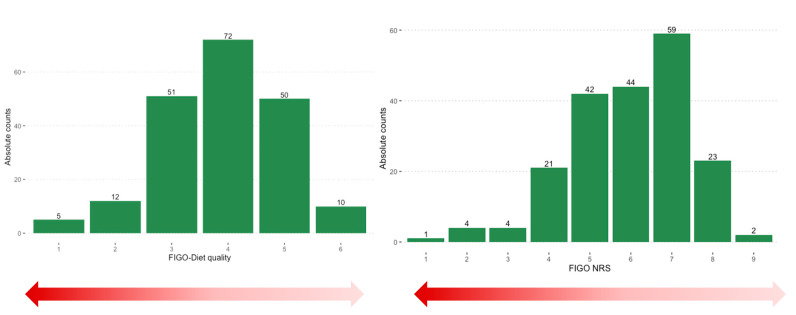
Histograms show participant distribution frequencies regarding the FIGO-Diet quality (**left**) and FIGO-NRS (**right**). FIGO—International Federation of Obstetrics and Gynecology; NRS—nutritional risk score.

A total of 30 participants (15%) exhibited 5 or more nutritional risk factors, as indicated by a total FIGO score of 1–4. Most women reported taking vitamin supplements (71.5%), and 52.5% were taking vitamin D ONS. A total of 6 out of 200 pregnant women (3%) reported special dietary requirements. One reported abstaining from all meat products; others reported nut allergies, ketogenic diet, lactose intolerance and irritable bowel syndrome, the avoidance of cow’s milk, and regular lent for religious reasons. Figure 2 presents the sample’s distribution frequency regarding the FIGO-Diet quality and FIGO-NRS scores.

Differences in the FIGO-NRS between participant subgroups are presented in Table 4. The only observed difference involved women with a low MUAC, indicative of undernutrition (<23 cm), who also exhibited a lower FIGO-NRS compared with the rest of the sample, suggesting greater nutritional risk.

### 3.2. Relationship between Maternal MUAC and FIGO-NRS

**Figure 3:**
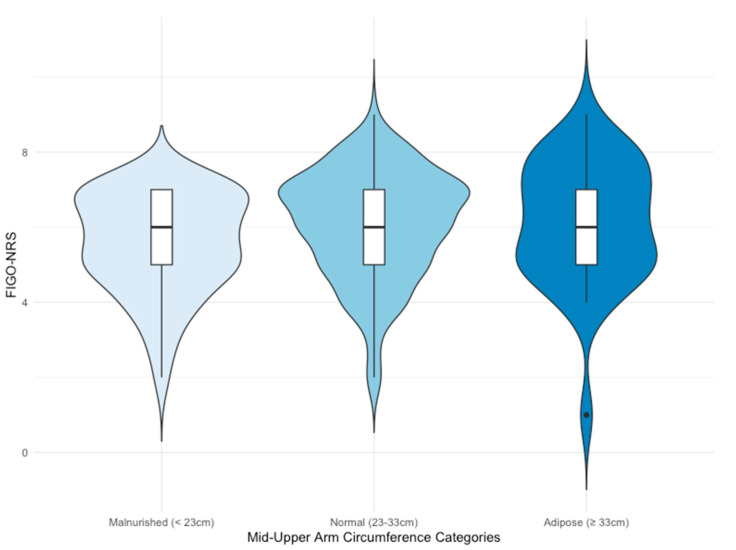
Association between FIGO-NRS and MUAC categories. FIGO—International Federation of Obstetrics and Gynecology; MUAC—middle-upper arm circumference; NRS—nutritional risk score.

Figure 3 presents the violin plots of FIGO-NRS between participants of different MUAC categories (undernourished, well-nourished, and over-nourished). Based on the Kruskal–Wallis test, no differences were noted in the FIGO-NRS between pregnant women belonging to distinct MUAC categories (*p* = 0.10).

### 3.3. Factors Affecting Diet Quality of Pregnant Women Based on the FIGO Nutrition Checklist

Table 5 details the univariable and multivariable binary logistic regression models examining the association of having a FIGO Diet Quality Score > 3. In the crude univariable model, being in the third trimester of pregnancy, taking vitamin D ONS, and being diagnosed with hypertensive disorders of pregnancy (HDP) or hyperemesis gravidarum were associated with greater chances of following a diet of better quality. In the multivariable model, only having hyperemesis gravidarum and receiving vitamin D ONS remained significant predictors of improved diet quality.

### 3.4. Factors Affecting the Nutritional Risk of Pregnant Women Based on the FIGO Nutrition Checklist

Table 6 details the univariable and multivariable binary logistic regression models examining the association between having a FIGO-NRS < 5 (higher nutritional risk compared with those having a FIGO-NRS ≥ 5) and other clinical factors associated with nutrition among pregnant women. In both models, being in the third trimester of pregnancy was the only factor associated with a lower nutritional risk.

## 4. Discussion

The present study used the FIGO nutrition checklist to evaluate diet quality and nutritional risk factors in pregnant women attending antenatal care in Greece. Based on the checklist, the results revealed that 99% of the participating pregnant women had at least one nutritional risk factor. The median FIGO diet quality score of the sample was 4.0 (3.0–5.0), with 95% of the participants responding negatively to at least one question, indicating the need to improve their diet quality. Women with hyperemesis gravidarum who received vitamin D ONS had increased odds of following a better-quality diet. A great proportion of the sample (36%) exhibited five or more nutritional risk factors, as indicated by a total FIGO-NRS < 5. Women with low MUAC, indicative of protein-energy malnutrition (PEM), exhibited more nutritional risk factors compared with the rest of the sample. On the other hand, being in the third trimester of pregnancy was associated with lower nutritional risk and, thus, better diet quality.

### 4.1. Diet Quality during Pregnancy

Many studies have reported suboptimal diet quality during pregnancy [8,16,17,38,39,40], as observed herein. Research in the USA has suggested that younger age, with lower education, more children, and greater pre-pregnancy BMI, is associated with poorer diet quality [40], although such associations are not confirmed herein. In parallel, being diagnosed with hyperemesis gravidarum and receiving vitamin D ONS were associated with the adoption of diets of better quality in the sample. Specific conditions, including hyperemesis gravidarum and low income, have previously been identified as effectors of diet quality during pregnancy [41]. In contrast, the intake of dietary supplements has also been related to a greater preoccupation with following a “healthier” diet during gestation [13]. Despite the quality of the diet, however, research suggests that due to nausea and vomiting, the amount of food consumed is significantly less in hyperemesis, with the majority of women failing to cover the recommended intakes for most nutrients [42].

In our sample, diet quality was improved and the number of nutritional risk factors was reduced in the third trimester of gestation. Previous Greek data revealed improved diet quality with ascending pregnancy trimesters [16]. Given that most pregnancies are unplanned, women are eager to improve their diet when pregnancy is confirmed in an effort to correct for possible previous unhealthy eating habits [13].

### 4.2. Nutritional Risk Factors during Pregnancy

In the present study, nearly all participants exhibited at least one nutritional risk factor, whereas 36% of the sample exhibited five or more nutritional risk factors, as indicated by a total FIGO score < 5. Similar findings have also been noted in South Africa, with 97.4% of the participants (pregnant and non-pregnant women) demonstrating at least one nutritional risk using the FIGO nutrition checklist [43]. Among the most common nutritional risk factors identified herein are the frequent consumption of packaged foods (59.5%), inadequate sun exposure (52%), and the inadequate intake of fruit, vegetables, and fish (43.5%). The consumption of ultra-processed foods indicates poor maternal diet quality [44], and according to a recent systematic review, it is also associated with adverse perinatal outcomes, including GDM and preeclampsia [45].

On the other hand, inadequate fruit and vegetable intake is associated with a greater risk for micronutrient deficiencies, lower GWG [46], lower neonatal birth weight [47,48,49] and length [49], a greater risk of giving birth to low birth weight (LBW) infants [47], an unfavorable infant gut microbiome [50], as well as greater odds of their offspring’s developmental delays [51]. Previous Greek data revealed an inadequate fruit and vegetable intake among pregnant women [16], indicating the need for nutrition education.

Inadequate sun exposure, hypovitaminosis D, and vitamin D deficiency (VDD) are common during pregnancy [52,53,54]. In parallel, despite being one of Europe’s sunniest countries, several studies have revealed a great prevalence of hypovitaminosis D and VDD in the Greek population [55]. A systematic review [56] of women from Mediterranean countries, including pregnant women, revealed a 23–90% deficiency prevalence with possible adverse consequences for maternal and neonatal health. Low vitamin D levels have been associated with a greater risk for preeclampsia, GDM, and bacterial vaginosis [57,58,59], preterm birth [57,58,60,61], giving birth to small-for-gestational-age (SGA) [58,61] and LBW infants [58], maternal and infant infections [57], the need for caesarian deliveries [57,58], lower offspring scores in mental and developmental tests [61], and many more. Among the risk factors for low vitamin D concentrations are less time spent outdoors, the use of sunscreens, lower skin exposure due to clothing choices, or avoidance of exposure to UVB radiation [54]. Nonetheless, in our sample, 52.5% of the participating women reported taking regular vitamin D ONS, indicating that for nearly 50% of the sample, inadequate sun exposure is potentially leveraged by the supplemental intake of vitamin D.

A recent study [43] also associated food insecurity with having more than three at-risk practices based on the FIGO nutrition checklist, indicating that difficulties in accessing healthy and nutritious food is an important issue for many women of reproductive age.

### 4.3. MUAC vs. the FIGO Nutrition Checklist

The WHO proposed the measurement of MUAC [62] for the early identification of PEM among pregnant women. Low maternal MUAC has been associated with an increased risk for LBW and stunting in the newborn [63,64]. In the present sample, 20.6% of the participants were identified as having PEM with the use of the MUAC measurement, a similar proportion to that observed among pregnant women residing in Ethiopia (21.8%) [65] and South Africa [66]. Several researchers have used maternal MUAC for the stratification of the nutritional status of pregnant women [65,66,67,68], and recently, its use as a fast tool, not requiring specific nutrition education, has been suggested [69]. Despite its ease of use and ability to identify PEM, it does not account for other nutritional risk factors, including vitamin D levels and fruit and vegetable intake.

As a result, although the WHO [62] proposed MUAC for identifying PEM in individual pregnant women, in our sample, participants with low MUAC exhibited greater FIGO-NRS; however, no differences were noted in the FIGO-NRS of women with high MUAC. In this manner, it is highly possible that dietary excess was not accounted for in the FIGO checklist [28], whereas, in parallel, other nutritional factors aside from PEM are not accounted for when measuring MUAC. In parallel, although a question regarding body weight and GWG exists in the checklist (Section 2), according to the FIGO [28], negative answers to any of the questions in Section 3 or Section 4 indicate a more detailed nutritional assessment may be required. Sections 1 and 2 are not included in the triage procedure. Furthermore, there is no “gold standard” for the assessment of nutritional risk among pregnant women that could be used for the validation of the tool, and for this, Tsoi and associates [36] only validated the FFQ part (Section 3) against typical diet quality indexes.

### 4.4. Other Screening Tools Proposed for the Identification of Nutritionally Vulnerable Pregnant Women

The first screening tool for the identification of nutritionally vulnerable pregnant women was established in the 80’s by Agnes Higgins [70]. The “Higgins method” was based on the dietary analysis of a 7-day food intake record (concerning protein and energy intake). Diet prescription was calculated according to individual requirements, including the additional pregnancy requirements, a corrective allowance for underweight and undernutrition, and a correction for nutritional stress (due to pernicious vomiting, recent pregnancy, poor obstetrical outcome, failure to gain 10 lbs by 20 weeks, and severe emotional stress), based on the formula developed by Agnes Higgins at the Montreal Diet Dispensary [70]. Although effective and considered by some as the gold standard for evaluating antenatal nutritional risk screening, it could only be applied by experienced dietitians, thus limiting its use by the other HPCs. In parallel, it requires much time from the part of the involved dietitians, as it does not only screen but also recommend dietary intake tailored for each participant.

Soon after, Kennedy and associates [71] proposed another screening tool that included maternal age, parity, pre-pregnancy weight-for-height, length of inter-conceptual periods, history of giving birth to LBW infants, and miscarriages, as well as a modified FFQ using four food groups (meat and meat alternates, milk, bread and cereal, and fruit and vegetables). This tool was not applied in research.

The Higgins method validated a shorter screening tool developed by Duquette and colleagues [72] based on 26 risk factors. Nineteen of these were non-dietary, including age, income, type of employment, smoking, drug use, drinking, closely spaced pregnancy, parity, previous LBW babies, previous abortions, illnesses, pre-gestational weight, GWG, pregnancy complications, gestational age, single/multiple pregnancies, serious emotional problems, an absent father, and overall support. The remaining seven regarded dietary factors, including the interval between meals and the frequency of consumption of six food groups for the estimation of protein intake: (i) milk and yogurt; (ii) bread, potatoes, and cereal products; (iii) fish, meat, poultry, and liver; (iv) cheese; (v) eggs; and (vi) nuts, legumes, and peanut butter. Unfortunately, this tool has not been used in the scientific literature.

More recently, Anaya-Prado and colleagues [73] adapted the Nutritional Risk Screening (NRS 2002) criteria developed by the European Society of Parenteral and Enteral Nutrition (ESPEN) [74,75] to obstetrical patients. In their analysis, however, the NRS was only associated with maternal morbidity, leaving out all the other important obstetrical and offspring outcomes.

### 4.5. The Use of the FIGO Nutrition Checklist

Table 7 details all the available primary research conducted to date using the FIGO Nutrition Checklist in women of reproductive age [27,43], pregnant women [30,36,43,76,77], and pre-pregnant women [30], as well as on HCPs [27,30,76]. An additional qualitative study is currently being conducted on women in healthy singleton pregnancies [78], without published results yet.

In Ireland [76], 80% of the pregnant women exhibited at least one nutritional risk, whereas in India, most were overweight/obese, and 1/3 exhibited low Hb concentrations [30]. Most women considered the checklist quick to complete [76] and acceptable [27]. Women also noted the usefulness of discussing nutrition as part of routine care rather than in a separate appointment [77]. Compared with other diet quality indexes, associations were noted with the total checklist score of pregnant women [36]. In parallel, the question on fruits/vegetables was associated with the consumption of fiber, vitamin C, and fruits and vegetables, as calculated from a food-frequency questionnaire (FFQ) [36]. On the other hand, the question regarding dairy intake was related to the maternal intake of Ca, milk, and dairy products, captured through FFQs [36].

From the HPCs perspective (Table 6), most of them considered the checklist acceptable [27] and simple to use [30] and would recommend it to colleagues for use in routine antenatal care [76]. For 1/5 of the HPCs, administering the checklist did not take more than 5 min [30], whereas, for the remaining HPCs, 5–10 min were adequate for its completion. Most OBGYNs found the checklist helped them discuss GWG and nutrition with more women than they normally would [76]. Barriers reported for the lack of implementation of the checklist included a lack of time, a lack of specific training for HCPs, and the need for non-stigmatizing communication with patients [27]. Collectively, the studies using the checklist indicate its feasibility and applicability in different countries, its ease of use by a diverse HCP audience, and its acceptability by both HCPs and women.

### 4.6. Strengths and Limitations of the Study

The present study is biased by its cross-sectional nature and the relatively small number of participants. In parallel, the checklist was developed to identify women in need of further nutritional assessment and education (based on the answers in Section 3 and Section 4); however, since a gold standard for the identification of nutritional risk in pregnant women has not yet been established, it cannot be validated for this use. Nonetheless, the checklist is extremely useful and serves its purpose since factors known to be associated with increased nutritional risk are incorporated in the tool, and its use by HCPs who are not experts in nutrition is ensured.

Of note, several other factors that affect nutritional status during gestation are not included in the checklist and could be considered in a revised version of the tool, including food insecurity and disordered eating behaviors. In parallel, equal weight is currently given to each item in Sections 3 and 4 of the checklist for those who wish to score nutritional risk and diet quality, as suggested by Tsoi and associates [36]. Attempts to quantify nutritional risk among pregnant women also weighted all food groups equally [70,71,72]. However, discrepancies exist even in the intake of single food groups. For example, are all dairy products equal? When questioning one’s frequency of daily dairy product intake, should we consider fortified, low-fat, and full-fat milk as equally nutritious? On the other hand, this is only important when an accurate diet history is pursued. These questions obviously can remain general when the intent is only to improve and facilitate triage for malnutrition from heterogeneous HPCs.

At the moment, no longitudinal study has evaluated the pregnancy outcomes associated with each of the nutritional risk factors included in the FIGO nutrition checklist, thus we cannot conclude on which factors may be more detrimental for the mother or the fetus. However, an ongoing Italian longitudinal study [78] is expected to shed light on this issue when data collection is complete and the results are published.

Last, the first section of the checklist could be improved, as a variety of possible answers were encountered that were not equally related to malnutrition risk, including abstaining from all meat products, have nut allergies, following a ketogenic diet, having lactose intolerance, having irritable bowel syndrome, avoiding cow’s milk, and practicing regular lent for religious reasons. In essence, what respondents may a consider restrictive dietary pattern does not always correspond to increased nutritional risk.

## 5. Conclusions

The FIGO nutrition checklist is a useful tool for everyday antenatal clinical practice, rapid and easy to use by most HCPs. It can aid in initiating conversations regarding GWG and nutrition and identifying the women who might require further nutritional assessment and counseling. The use of the tool in a population of pregnant Greek women revealed that a high proportion of the sample carried several nutritional risks, while most adopted suboptimal diets. The results of the present study showed that the use of the FIGO nutrition checklist is feasible in the Greek healthcare setting, but future research should also focus on evaluating its comprehensiveness and acceptability by Greek HPCs.

## Figures and Tables

**Table 1:** Baseline characteristics of the participating pregnant women (*n* = 200).

	Characteristics	Value *
General	Age (years)	32 (27–36)
	Smoking Yes/No (*n*)	51 (25.5%)/149 (74.5%)
Anthropometry	Body weight (kg)	75.0 (64.0–86.6)
	BMI (kg/m^2^)	27.3 (23.9–31.8)
	Pre-gravid body weight (kg)	56.0 (68.0–78.0)
	Pre-gravid BMI (kg/m^2^)	24.2 (21.4–29.2)
	Exceeding IOM GWG recommendations Yes/No (*n*)	33 (16.5%)/167 (83.5%)
	MUAC	26.5 (23.0–31.0)
	Undernourished/adequacy nourished/obese based on the MUAC (*n*)	41 (20.6%)/124 (62.3%)/34 (17.1%)
Societal factors	Education attained none/secondary/tertiary (*n*)	10 (5.0%)/85 (43.0%)/105 (52.0%)
	Marital status Married/Single/Widowed (*n*)	170 (85.0%)/29 (14.5%)/1 (0.5%)
	Immigrant status Yes/No (*n*)	12 (6.0%)/188 (94%)
Obstetrical factors	Gravidity 1st/2nd/3rd/4th/6th	83 (42%)/81 (40%)/26 (13%)/9 (4.5%)/1 (0.5%)
	Number of offspring 0/1/2/3/5 (*n*)	88 (44%)/77 (38.5%)/24 (12%)/10 (5%)/1 (0.5%)
	Singleton/twin pregnancies (*n*)	198 (99%)/2 (1%)
	Method of conception natural/ART (*n*)	177 (89%)/22 (11%)
	Trimester of pregnancy 1st/2nd/3rd (*n*)	45 (22.5%)/71 (35.5%)/84 (42.0%)
	GDM diagnosis Yes/No (*n*)	43 (21.5%)/157 (78.5%)
	Hyperemesis gravidarum diagnosis Yes/No (*n*)	33 (16.5%)/167 (83.5%)
	HDP diagnosis Yes/No (*n*)	7 (3.5 %)/193 (96.5 %)
	Preeclampsia diagnosis Yes/No (*n*)	5 (2.5%)/195 (97.5%)
Annual household income (€)	<5 K/5 K–10 K/10 K–15 K/≥15 K (*n*)	30 (15.1%)/71 (35.7%)/61 (30.7%)/37 (18.6%)

ART—assisted reproductive technologies; BMI—body mass index; GDM—gestational diabetes mellitus; GWG—gestational weight gain; HDP—hypertensive disorders of pregnancy; IQR—inter-quartile range; IOM—Institute of Medicine [34]; K—1000; MUAC—middle-upper arm circumference. * Data are presented as counts (*n*) with their respective percentages, or as medians with their respective IQR.

**Table 2:** Participant answers in the FIGO Nutrition Checklist [28] (*n* = 200).

FIGO Nutrition Checklist Components		Positive Answers	Score *
**Quality of diet**	Meat/poultry consumption (2–3/week)	172 (86%)	
	Fruit and vegetable consumption (2–3/day)	113 (56.5%)	
	Fish consumption (1–2 times/week)	113 (56.5%)	
	Dairy products intake (daily)	165 (82.5%)	
	Whole grain carbohydrates intake (≥1/day)	136 (68%)	
	Packaged foods intake (≤5 times/week)	81 (40.5%)	
	Pregnant women reporting ≥ one negative answer	190 (95%)	
	Pregnant women reporting no negative answers	10 (5%)	
**FIGO–Diet Quality Score ^†^**			**4 (3–5)**
**Other nutritional aspects**	Folic acid ONS	146 (73%)	
	Regular sun exposure	96 (48%)	
	Normal Hb concentration (≥110 g/L)	172 (86%)	
	Pregnant women reporting ≥ one negative answer	199 (99%)	
	Pregnant women reporting no negative answers	2 (1%)	
**FIGO–NRS (overall nutritional status) ^‡^**			**6 (5–7)**

FIGO—International Federation of Obstetrics and Gynecology; Hb—hemoglobin; IQR—interquartile range; NRS—nutritional risk score; ONS—oral nutrient supplementation. * median with respective IQR; ^†^ score ranges between 0 and 6, with higher scores indicative of better diet quality [36]. ^‡^ score ranges between 0 and 9, with lower scores indicating greater nutritional risk.

**Table 3:** Participant positive answers in the FIGO Nutrition Checklist [28] and scores, according to trimester of pregnancy (*n* = 200).

	FIGO Nutrition Checklist Components	1st Trimester (*n* = 45)	2nd Trimester (*n* = 71)	3rd Trimester (*n* = 84)	*p*-Value
**Quality of diet**	Meat/poultry consumption (2–3/week)	39 (87%)	56 (79%)	77 (92%)	0.07 ^∞^
	Fruit and vegetable consumption (2–3/day)	26 (58%)	37 (52%)	50 (59%)	0.64 ^∞^
	Fish consumption (1–2 times/week)	27 (60%)	42 (59%)	44 (52%)	0.60 ^∞^
	Dairy products intake (daily)	35 (78%)	56 (79%)	74 (88%)	0.21 ^∞^
	Whole grain carbohydrates intake (≥1/day)	27 (60%)	46 (65%)	63 (75%)	0.17 ^∞^
	Packaged foods intake (≤5 times/week)	16 (36%)	28 (39%)	37 (44%)	0.63 ^∞^
	**FIGO–Diet Quality Score ^†^**	4 (3–4) *	4 (3–5) *	4 (3–5) *	0.12 ^*f*^
**Other nutritional** **aspects**	Folic acid ONS	24 (53%)	54 (76%)	68 (81%)	0.003 ^∞^
	Regular sun exposure	19 (42%)	29 (41%)	48 (57%)	0.09 ^∞^
	Normal Hb concentration (≥110 g/L)	26 (58%)	65 (91%)	81 (96%)	<0.001 ^*f*^
	**FIGO–NRS (overall nutritional status) ^‡^**	5 (4–7) *	6 (5–7) *	7 (6–7) *	<0.001 ^*f*^

FIGO—International Federation of Obstetrics and Gynecology; Hb—hemoglobin; IQR—interquartile range; NRS—nutritional risk score; ONS—oral nutrient supplementation. * median with respective IQR; ^†^ score ranges between 0 and 6, with higher scores indicative of better diet quality [36]. ^‡^ score ranges between 0 and 9, with lower scores indicating greater nutritional risk; ^∞^ According to the chi-squared test; ^ƒ^ According to the Kruskal–Wallis test for non-parametric data.

**Table 4:** Differences in the FIGO-NRS between subgroups of participants (*n* = 200).

Variable	Yes		No		*p*-Value
	*n*	NRS Score	*n*	NRS Score	
Immigrant status	*n* = 12	6.0 (4.75–6.0)	*n* = 188	6.0 (5.0–7.0)	0.23
GDM	*n* = 43	7.0 (5.0–7.0)	*n* = 157	6.0 (5.0–7.0)	0.19
HDP	*n* = 7	7.0 (5.5–8.0)	*n* = 193	6.0 (5.0–7.0)	0.25
Hyperemesis gravidarum	*n* = 33	6.0 (5.0–7.0)	*n* = 167	6.0 (5.0–7.0)	0.45
Primiparas	*n* = 83	6.0 (5.0–7.0)	*n* = 117	6.0 (5.0–7.0)	0.82
Twin pregnancy	*n* = 2	5.5 (4.8–6.3)	*n* = 198	6.0 (5.0–7.0)	0.66
Low MUAC (<23 cm) ^†^	*n* = 41	6.0 (5.0–7.0)	*n* = 158	6.0 (5.0–7.0)	0.03
High MUAC (≥33 cm) ^†^	*n* = 34	6.0 (5.0–7.0)	*n* = 165	6.0 (5.0–7.0)	0.51
Exceeding GWG recommendations *	*n* = 33	6.0 (5.0−7.0)	*n* = 167	6.0 (5.0–7.0)	0.87
ART conception	*n* = 22	7.0 (5.0–7.0)	*n* = 177	6.0 (5.0–7.0)	0.28

ART—assisted reproductive technologies; FIGO—International Federation of Obstetrics and Gynecology; GDM—gestational diabetes mellitus; GWG—gestational weight gain; HDP—hypertensive disorders of pregnancy; IOM—Institute of Medicine; MUAC—middle-upper arm circumference; NRS—nutritional risk score; WHO—World Health Organization. * based on the IOM recommendations [34]; ^†^ based on the WHO cut-offs.

**Table 5:** Univariable and multivariable binary logistic regression models examining the association of having a FIGO Diet Quality Score > 3 (a healthier quality of diet) with factors affecting diet quality among pregnant women (*n* = 200).

Variables ^†^		Univariable Model				Multivariable Models							
		OR	95% CI		*p*-Value	aOR	95% CI		*p*-Value	aOR	95% CI		*p*-Value
			Lower	Upper			Lower	Upper			Lower	Upper	
Gravidity	Primiparas	1	-	-	-	1	-	-	-				
	Multiparas	0.98	0.54	1.78	0.95	1.09	0.52	2.28	0.82				
Education attained	None	1	-	-	-	1	-	-	-				
	Secondary	1.00	0.26	3.81	1.00	1.23	0.27	5.74	0.79				
	Tertiary	1.67	0.44	6.33	0.45	2.29	0.47	11.26	0.31				
Annual income (€)	<5 K	1	-	-	-	1	-	-	-				
	5 K–10 K	0.79	0.31	1.98	0.61	0.65	0.23	1.89	0.43				
	10 K–15 K	0.95	0.37	2.45	0.91	0.62	0.20	1.98	0.42				
	>15 K	0.70	0.25	1.96	0.50	0.32	0.08	1.23	0.10				
Method of conception	Natural	1	-	-	-	1	-	-	-				
	ART	1.10	0.43	2.84	0.85	0.79	0.25	2.49	0.69				
Trimester of gestation	First	1	-	-	-	1	-	-	-				
	Second	0.85	0.39	1.84	0.68	0.60	0.24	1.48	0.27				
	Third	1.38	0.64	2.99	0.42	1.57	0.59	4.17	0.36				
BMI (kg/m^2^)		0.98	0.93	1.03	0.39	0.97	0.90	1.05	0.46				
GDM	No	1	-	-	-	1	-	-	-				
	Yes	1.09	0.53	2.23	0.82	1.00	0.42	2.39	1.00				
HDP	No	1	-	-	-	1	-	-	-				
	Yes	1.88	1.40	2.53	0.29	4.89	0.38	62.37	0.22				
Hyperemesis gravidarum	No	1	-	-	-	1	-	-	-	1	-	-	-
	Yes	2.66	1.04	6.79	0.04	4.35	1.35	14.04	0.01	2.66	1.03	6.83	0.04
Vitamin D ONS	No	1	-	-	-	1	-	-	-	1	-	-	-
	Yes	1.67	0.92	3.01	0.09	2.11	1.05	4.23	0.04	1.67	0.92	3.03	0.09
Other ONS	No	1	-	-	-	1	-	-	-				
	Yes	0.96	0.50	1.84	0.90	0.71	0.33	1.55	0.40				
Smoking	No	1	-	-	-	1	-	-	-				
	Yes	0.74	0.38	1.42	0.36	1.06	0.49	2.27	0.89				
Age (years)		1.01	0.96	1.06	0.73	1.01	0.94	1.08	0.85				
Immigrant status	No	1	-	-	-	1	-	-	-				
	Yes	0.71	0.22	2.31	0.57	0.31	0.07	1.29	0.11				
Excessive GWG *	No	1	-	-	-	1	-	-	-				
	Yes	0.76	0.35	1.63	0.48	0.65	0.23	1.82	0.41				

aOR—adjusted odds ratio; ART—assisted reproductive technologies; BMI—body mass index; CI—confidence intervals; FIGO—International Federation of Obstetrics and Gynecology; GDM—gestational diabetes mellitus; GWG—gestational weight gain; HDP—hypertensive disorders of pregnancy; HG—hyperemesis gravidarum; IOM—Institute of Medicine; K—1000; ONS—oral nutrient supplementation; OR—odds ratio. * based on the IOM recommendations [34]; ^†^ variables with no counts were excluded from the *p*-value calculations.

**Table 6:** Univariable and multivariable binary logistic regression models examining the association of having a FIGO-NRS < 5 (greater nutritional risk compared with FIGO-NRS ≥ 5) with clinical factors associated with nutrition among pregnant women (*n* = 200).

Variables ^†^		Univariable Model				Multivariable Model			
		OR	95% CI		*p*-Value	aOR	95% CI		*p*-Value
			Lower	Upper			Lower	Upper	
Gravidity	Primiparas	1	-	-	-	1	-	-	-
	Multiparas	0.915	0.418	2.005	0.825	1.048	0.354	3.100	0.932
Education attained	None	1	-	-	-	1	-	-	-
	Secondary	0.627	0.147	2.670	0.528	0.805	0.158	4.104	0.794
	Tertiary	0.219	0.048	0.995	0.049	0.328	0.056	1.935	0.218
Annual income (€)	<5 K	1	-	-	-	1	-	-	-
	5 K–10 K	0.982	0.337	2.860	0.974	1.003	0.293	3.438	0.996
	10 K–15 K	0.281	0.073	1.085	0.066	0.328	0.068	1.585	0.166
	>15 K	0.625	0.170	2.292	0.478	1.140	0.229	5.668	0.873
Method of conception	Natural	1	-	-	-	1	-	-	-
	ART	0.917	0.253	3.321	0.895	1.395	0.295	6.593	0.674
Pregnancy trimester	First	1	-	-	-	1	-	-	-
	Second	0.501	0.205	1.225	0.130	0.654	0.229	1.870	0.429
	Third	0.156	0.051	0.473	0.001	0.174	0.047	0.648	0.009
BMI (kg/m^2^)		0.975	0.906	1.050	0.493	0.996	0.898	1.105	0.942
GDM	No	1	-	-	-	1	-	-	-
	Yes	1.133	0.450	2.850	0.791	2.122	0.618	7.292	0.232
HDP	No	1	-	-	-	1	-	-	-
	Yes	0.943	0.109	8.120	0.957	0.719	0.056	9.311	0.801
Hyperemesis gravidarum	No	1	-	-	-	1	-	-	-
	Yes	0.748	0.243	2.306	0.613	0.400	0.092	9.311	0.223
Smoking	No	1	-	-	-	1	-	-	-
	Yes	1.573	0.682	3.631	0.288	1.038	0.384	2.808	0.942
Age (years)		0.963	0.901	1.03	0.272	0.992	0.900	1.094	0.874
Immigrant status	No	1	-	-	-	1	-	-	-
	Yes	1.988	0.506	7.812	0.325	0.697	0.144	3.374	0.459
Excessive GWG *	No	1	-	-	-	1	-	-	-
	Yes	0.519	0.148	1.821	0.305	0.697	0.144	3.374	0.653

aOR—adjusted odds ratio; ART—assisted reproductive technologies; BMI—body mass index; CI—confidence intervals; FIGO—International Federation of Obstetrics and Gynecology; GDM—gestational diabetes mellitus; GWG—gestational weight gain; HDP—hypertensive disorders of pregnancy; IOM—Institute of Medicine; K—1000; NRS—nutritional risk score; ONS—oral nutrient supplementation; OR—odds ratio. * based on the IOM recommendations [34]; ^†^ variables with no counts were excluded from the *p*-value calculations.

**Table 7:** Primary research using the FIGO Nutrition Checklist.

First Author	Origin	Study Design	Participants	Participant Age (Years)	Results
Killeen [76]	Ireland	Quantitative, cross-sectional	*n* = 105 pregnant women	33.3 ± 4.2	Most women (80.0%) answered at least one question negatively, indicating a potential nutritional risk. Nearly all women (99.0%) considered the checklist quick to complete.
		Qualitative, cross-sectional	*n* = 3 OBGYNs	NR	All OBGYNs agreed that nutrition discussions are important during gestation, but two of them considered the topics of nutrition and GWG difficult to initiate in routine care. Only one OBGYN felt confident discussing nutrition. All OBGYNs agreed that the checklist helped discuss GWG and nutrition with more women than normally. Two out of three OBGYNs would recommend using the checklist in clinical practice.
Killeen [77]	Ireland	Qualitative, cross-sectional	*n* = 10 pregnant women	NR	The first trimester was identified as the greatest priority for using the checklist. The convenience of having nutrition addressed as part of routine care rather than a separate appointment was also noted.
Soepnel [43]	South Africa	Mixed-methods, cross-sectional	*n* = 387 pregnant women (*n* = 96 pregnant and *n* = 291 non-pregnant) with overweight/obesity	24.0 (21.8– 26.3)	The majority (97.4%) answered “no” to ≥1 diet quality question, indicative of an at-risk dietary practice. Food insecurity was positively associated with ≥3 at-risk practices (OR 1.87).
Tsoi [36]	China	Quantitative, cross-sectional	*n* = 156 pregnant women	32.7 ± 3.9	The checklist score was associated with several diet quality indicators (DASH, DQI-I, and MDS). The question on fruit/vegetables was associated with the consumption of fiber, vitamin C, and fruit and vegetables, as calculated from the FFQ. The question on dairy intake was related to the intake of Ca, milk, and dairy products captured through the FFQ.
Jacob * [27]	UK	Quantitative, cross-sectional	*n* = 251 women in the reproductive age	18–45	The concept and content of the checklist were acceptable to both women and HCPs (>80% in both groups). Several barriers reduce the checklist’s inmplementation, including a lack of time, training for HCPs, and the need for un-stigmatizing communication. Both groups considered routine nutrition discussions important; however, nutrition does not appear to be regularly discussed in the UK.
		Qualitative, cross-sectional	*n* = 47 HCPs	NR	
Divakar [30]	India	Quantitative, cross-sectional	*n* = 714 pregnant and prepregnant women	18–45	A large proportion (48%) of women were overweight/obese and 33% had low Hb concentrations. Greater Hb concentrations were associated with ONS intake.
			*n* = 50 HCPs	NR	Among the HCPs, 18% considered using the checklist “somewhat difficult”, while 82% found it “simple”. Furthermore, 26% of the respondents spent only 5’ to administer the checklist, while the rest (72%) spent 5–10’. The majority (62%) of HPCs reported that before introducing the checklist, a dietary history was taken as part of routine care and argued that the information collected through the checklist aids in the delivery of recommendations regarding GWG and nutritional deficiencies (90% of HPCs).

DASH—Dietary Approaches to Stop Hypertension; DQI-I—Diet Quality Index International; FIGO—International Federation of Obstetrics and Gynecology; GWG—gestational weight gain; HCPs—health care practitioners; Hb—hemoglobin; MDS—Mediterranean Diet Score; NR—not reported; OBGYN—obstetrician-gynecologist; ONS—oral nutrient supplementation; OR—odds ratio; * the checklist was modified based on UK dietary guidelines and clinical recommendations for obstetrics and antenatal care suggested by the National Institute for Health and Care Excellence.

## Data Availability

Data are provided upon request from the authors.

## Supplementary Materials

The following supporting information can be downloaded at: https://www.mdpi.com/article/10.3390/nu15092019/s1, Translation of the FIGO Nutrition Checklist in the Greek language.
