# Supplementary material for: Diet Quality and Nutritional Risk Based on the FIGO Nutrition Checklist among Greek Pregnant Women: A Cross-Sectional Routine Antenatal Care Study
Source: Nutrients. 2023 Apr 22;15(9):2019. doi: 10.3390/nu15092019 (PMC10181041; doi:10.3390/nu15092019)
Supplement: Supplementary file 1 [file nutrients-15-02019-s001.zip › nutrients-2315184-supplementary.pdf]

Διεθνής Ομοσπονδία Μαιευτικής και Γυναικολογίας

Διατροφική λίστα ελέγχου (nutrition checklist) πριν την κύηση και κατά την αρχόμενη κύηση.

Η καλή θρέψη της μητέρας πριν και κατά την διάρκεια της κύησης αποτελεί σημαντική προϋπόθεση για την καλή υγεία της ίδιας και του μωρού της. Η παρακάτω λίστα ελέγχου (checklist) σχεδιάστηκε ώστε να συμπληρώνεται από γυναίκες σε συνεργασία με τους/τις επαγγελματίες υγείας, ούτως ώστε να αξιολογηθεί εάν η διατροφική πρόσληψή τους είναι επαρκής. Επιμέρους στόχο έχει να καταδείξει στους επαγγελματίες υγείας τους τομείς της διατροφής της εξεταζόμενης που επιδέχονται βελτίωσης (εφόσον αυτό κρίνεται απαραίτητο).

Οι παρακάτω ερωτήσεις πρέπει να συμπληρωθούν από την γυναίκα, σε συνεργασία με τον επαγγελματία υγείας της:

1) Έχετε κάποια ιδιαίτερη διατροφική απαίτηση (π.χ. χορτοφαγία, αυστηρή χορτοφαγία/vegan, τροφικές αλλεργίες, κ.α.); Εάν ναι, αναφέρετε τις παρακάτω:

……

………

2) Ποιο είναι:

α. Το σωματικό σας βάρος: …….. (σε κιλά)
β. Το ύψος σας: ……….. (σε μέτρα)

γ. (*Να συμπληρωθεί από τον/την επαγγελματία υγείας*): Για να υπολογίσετε το δείκτη μάζας σώματος, διαιρέστε το βάρος (σε κιλά) με το ύψος (σε μέτρα) και ξανά με το ύψος σε μέτρα.

Ο δείκτης μάζας σώματός σας είναι ……….. kg/m^2.

3) Ποιότητα διατροφής:

i) Καταναλώνετε κρέας ή πουλερικά 2–3 φορές την εβδομάδα; Ναι ☐ Όχι ☐

ii) Καταναλώνετε συνήθως περισσότερες από 2–3 μερίδες φρούτων ή λαχανικών καθημερινά; Ναι ☐ Όχι ☐

iii) Καταναλώνετε ψάρι τουλάχιστον 1–2 φορές την εβδομάδα; Ναι ☐ Όχι ☐

iv) Καταναλώνετε γαλακτοκομικά προϊόντα όπως γάλα, τυρί, γιαούρτι, καθημερινά; Ναι ☐ Όχι ☐

v) Καταναλώνετε τροφές με υδατάνθρακες ολικής αλέσεως (π.χ. μαύρο ψωμί, ζυμαρικά ολικής αλέσεως, καστανό ρύζι) τουλάχιστον μια φορά την ημέρα; Ναι ☐ Όχι ☐

vi) Καταναλώνετε συσκευασμένα σνακ, κέικ, αρτοσκευάσματα ή ποτά με ζάχαρη σε συχνότητα **μικρότερη** από 5 φορές την εβδομάδα; Ναι ☐ Όχι ☐

4) Λοιπές ερωτήσεις:

i) Εφόσον είστε έγκυος, λάβατε/λαμβάνετε συμπληρώματα φυλλικού οξέος πριν και στην αρχή της κύησης (πρώτες 12 εβδομάδες); Ναι ☐ Όχι ☐

ii) Εκτίθεστε τακτικά στον ήλιο (πρόσωπο, άνω άκρα, χέρια) για τουλάχιστον 10–15 λεπτά ημερησίως; Ναι ☐ Όχι ☐

iii) Έχουν ελεγχθεί τα επίπεδα αιμοσφαιρίνης (ή επίπεδα σιδήρου) στο αίμα σας; Ναι ☐ Όχι ☐
(*Να συμπληρωθεί από τον/την επαγγελματία υγείας*): Εάν ναι, είναι η τιμή μεγαλύτερη από 110 g/L; Ναι ☐ Όχι ☐
Εισάγετε την τιμή: ……………..

Εφόσον έχετε απαντήσει Όχι σε οποιαδήποτε ερώτηση των τμημάτων 3 ή 4, θα πρέπει η διατροφική σας πρόσληψη να αξιολογηθεί με μεγαλύτερη λεπτομέρεια.

Περαιτέρω πληροφορίες για επαγγελματίες υγείας:

Πρόθεση είναι αυτό το έγγραφο να προσαρμοστεί στο πλαίσιο της χώρας στην οποία χρησιμοποιείται.

1. Οι φυσιολογικές τιμές του δείκτη μάζας σώματος (ΔΜΣ) αφορούν συνήθως τιμές μεταξύ 18.5–25 kg/m^2 σε σχέση με την ηλικία και τη γεωγραφική περιοχή διαβίωσης.
2. Για γυναίκες που δε βρίσκονται σε κύηση, παρέχετε συμβουλές για την επίτευξη ιδανικού σωματικού βάρους πριν την τεκνοποίηση.

   * Ενημερώστε τις γυναίκες σε κύηση για τους προτεινόμενους στόχους πρόσληψης βάρους ανάλογα με το ΔΜΣ τους πριν την κύηση (Πίνακας δεξιά). Λαμβάνετε πάντοτε υπόψη τις εθνικές κατευθυντήριες οδηγίες.
3. Μέρος 3, i: αξιολογείται η πρόσληψη βιταμίνης Β12, σιδήρου και πρωτεϊνών.
4. Μέρος 3, ii: αξιολογείται η πρόσληψη αντιοξειδωτικών, μικροθρεπτικών συστατικών και φυτικών ινών.

ΔΜΣ προ κύησης	Συνολική πρόσληψη βάρους (kg)	Ρυθμός πρόσληψης βάρους στο $2^{ο}$ και $3^{ο}$ τρίμηνο (kg/εβδομάδα)
**Υποθρεψία < 18,5 kg/m^2**	12,5 – 18	0,51 (0,44 – 0,58)
**Φυσιολογικό βάρος 18,5 – 24,9 kg/m^2**	11,5 – 16	0,42 (0,35 – 0,50)
**Υπερβάλλον βάρος 25,0 – 29,9 kg/m^2**	7 – 11,5	0,28 (0,23 – 0,33)
**Παχυσαρκία >30 kg/m^2**	5 – 9	0,22 (0,17 – 0,27)

Από τις οδηγίες του Institute of Medicine που εκδόθηκαν το 2009 https://nap.nationalacademies.org/resource/12584/Report-Brief---Weight-Gain-During-Pregnancy.pdf

5. Μέρος 3, iii: αξιολογείται η πρόσληψη ω-3/ω-6 πολυακόρεστων λιπαρών οξέων, βιταμίνη D και ιωδίου
6. Μέρος 3, iv: Αν η εξεταζόμενη απαντήσει **Όχι** σε αυτό ερώτημα, συνιστάται η υποκατάσταση ασβεστίου.
7. Μέρος 3, v και vi: Αν η απάντηση ήταν **Όχι**, συμβουλεύστε την εξεταζόμενη να αυξήσει την πρόσληψη υδατανθράκων ολικής αλέσεως και να ελαττώσει την κατανάλωση επεξεργασμένων υδατανθράκων.
8. Μέρος 4, i: Αν δε λαμβάνεται φυλλικό οξύ, συστήστε τη λήψη συμπληρωμάτων φυλλικού οξέος.
9. Μέρος 4, ii: Αν η εξεταζόμενη δεν εκτίθεται επαρκώς στον ήλιο ή έχει σκούρο χρώμα δέρματος, συνιστάται η συμπληρωματική χορήγηση βιταμίνης D.
10. Μέρος 4, iii: Αν τα επίπεδα αιμοσφαιρίνης είναι μικρότερα από 110 g/L συνιστάται η υποκατάσταση σιδήρου. Τα κατώτερα επίπεδα αιμοσφαιρίνης μπορεί να διαφέρουν μεταξύ γεωγραφικών περιοχών.
11. Οι επαγγελματίες υγείας θα πρέπει να εξετάζουν τυχόν τρόφιμα που είναι διαθέσιμα στη χώρα τους, τα οποία δε θεωρούνται ως ασφαλή προς κατανάλωση κατά τη διάρκεια της εγκυμοσύνης.
12. Εκτός από τις ερωτήσεις που υπάρχουν στο ερωτηματολόγιο, οι επαγγελματίες υγείας θα πρέπει να αξιολογήσουν και να παρέχουν συμβουλευτική εάν υπάρχουν άλλες μη ασφαλείς πτυχές του τρόπου ζωής των ασθενών, όπως το κάπνισμα, το αλκοόλ, η χρήση ναρκωτικών ή έλλειψη σωματικής άσκησης.

Πριν την κύηση και στο σχεδιασμό της κύησης		
**Εμπλεκόμενοι επαγγελματίες**	**Παράμετροι ελέγχου**	**Σημεία διαλόγου**
• Κοινωνικοί λειτουργοί<br>• Διατροφολόγοι<br>• Γενικοί/οικογενειακοί ιατροί<br>• Μαιευτήρες-Γυναικολόγοι<br>• Μαίες	• Σύνθεση δίαιτας<br>• Σωματική δραστηριότητα<br>• Ύψος, βάρος, ΔΜΣ<br>• Κίνδυνος παχυσαρκίας – περίμετρος μέσης (+ λοιπές ανθρωπομετρικές παράμετροι)<br>• Αναιμία<br>• Κίνδυνος συγκεκριμένων διατροφικών προβλημάτων<br>- Φυλλικό οξύ<br>- Σίδηρος<br>- Ασβέστιο<br>- Βιταμίνη Β12<br>- Βιταμίνη D<br>- Ιώδιο<br>- Ψευδάργυρος<br>- Πολυακόρεστα λιπαρά οξέα	• Σημασία υγιεινής διατροφής και άσκησης<br>• Προβλήματα καθιστικής συμπεριφοράς (π.χ. χρόνος μπροστά σε οθόνη)<br>• Συμπεριφορά και έκθεση κινδύνου<br>- Κάπνισμα, αλκοόλ, ναρκωτικές ουσίες<br>- Περιβαλλοντικές τοξίνες<br>• Πρώιμη διάγνωση και θεραπεία χρόνιων νόσων<br>• Υποκατάσταση θρεπτικών συστατικών:<br>- Φυλλικό οξύ 400 μg/ημέρα<br>- Άλλα θρεπτικά συστατικά (σίδηρος, ιώδιο, βιταμίνη Β12)

Κατά τη διάρκεια της κύησης		
**Εμπλεκόμενοι επαγγελματίες**	**Παράμετροι ελέγχου**	**Σημεία διαλόγου**
• Κοινωνικοί λειτουργοί<br>• Διατροφολόγοι<br>• Γενικοί/οικογενειακοί ιατροί<br>• Μαιευτήρες-Γυναικολόγοι<br>• Μαίες	• Σύνθεση δίαιτας<br>• Σωματική δραστηριότητα<br>• Ύψος, βάρος, ΔΜΣ<br>• Κίνδυνος παχυσαρκίας – περίμετρος μέσης (+ λοιπές ανθρωπομετρικές παράμετροι)<br>• Πρόσληψη βάρους στην κύηση<br>• Αρτηριακή πίεση<br>• Αναιμία<br>• Κίνδυνος συγκεκριμένων διατροφικών προβλημάτων<br>*Πρώτο τρίμηνο*<br>- Φυλλικό οξύ<br>- Βιταμίνη Β12<br>- Ιώδιο<br>- Πολυακόρεστα λιπαρά οξέα<br>*Δεύτερο και τρίτο τρίμηνο*<br>- Σίδηρος, ιώδιο, ψευδάργυρος, χαλκός, ασβέστιο<br>- Φυλλικό, βιταμίνες Β, D<br>- Ενέργεια (+450 kcal/ημέρα)<br>- Ιώδιο<br>- Πολυακόρεστα λιπαρά οξέα	• Διατροφική συμβουλευτική<br>• Ασφαλή επίπεδα άσκησης<br>• Χρόνος καθιστικής ζωής<br>• Συμπεριφορά και έκθεση κινδύνου<br>- Κάπνισμα, αλκοόλ, ναρκωτικές ουσίες<br>- Πηγές τροφιμογενών λοιμώξεων<br>• Πρώιμη διάγνωση και θεραπεία επιπλοκών της κύησης (σακχαρώδης διαβήτης της κύησης, αρτηριακή πίεση)<br>• Υποκατάσταση θρεπτικών συστατικών:<br>- Φυλλικό οξύ 400 μg/ημέρα<br>- Σίδηρος 30–60 mg/ημέρα<br>- Άλλα θρεπτικά συστατικά (ιώδιο, βιταμίνη Β12, D)

Αυτό το έγγραφο, συμπεριλαμβανομένου του παραπάνω πίνακα, βασίζεται στις συστάσεις της Διεθνούς Ομοσπονδίας Γυναικολογίας και Μαιευτικής σχετικά με τη διατροφή στην εφηβεία, πριν την τεκνοποίηση και κατά τη διάρκεια της εγκυμοσύνης: “Think Nutrition First”, Hanson M et al, 2015, πρόσβαση μέσω του συνδέσμου http://obgyn.onlinelibrary.wiley.com/hub/issue/10.1002/ijgo.2015.131.issue-S4/

Η FIGO είναι μια μη κυβερνητική οργάνωση που ειδικεύεται στη διασφάλιση της βελτίωσης της υγείας των γυναικών σε όλο τον κόσμο. Είναι ο μόνος παγκόσμιος οργανισμός που εκπροσωπεί εθνικές εταιρίες μαιευτήρων και γυναικολόγων και συνεργάζεται με άλλους επαγγελματίες υγείας και οργανισμούς για να εξασφαλίσει την καλύτερη δυνατή υγεία για τις γυναίκες του κόσμου.
